# Community health worker-based mobile health (mHealth) approaches for improving management and caregiver knowledge of common childhood infections: A systematic review

**DOI:** 10.7189/jogh.10.020438

**Published:** 2020-12

**Authors:** Hana Mahmood, Brian Mckinstry, Saturnino Luz, Karen Fairhurst, Sumaira Nasim, Tabish Hazir, RESPIRE Collaboration

**Affiliations:** 1 Maternal, Neonatal and Child Health Research Network (MNCHRN), Pakistan; 2 NIHR Global Health Research Unit on Respiratory Health (RESPIRE), Usher Institute, the University of Edinburgh, Edinburgh, UK

## Abstract

**Background:**

Children in lower middle-income countries (LMICs) are more at risk of dying, than those in High Income Countries (HICs), due to highly prevalent deadly yet preventable childhood infections. Alongside concerns about the incidence of these infections, there has been a renewed interest in involving community health workers (CHWs) in various public health programs. However, as CHWs are increasingly asked to take on different tasks there is a risk that their workload may become unmanageable. One solution to help reduce this burden is the use of mobile health (mHealth) technology in the community through behaviour change. Considering there are various CHWs based mHealth approaches on illness management and education, therefore, we aimed to appraise the available literature on effectiveness of these mHealth approaches for caregivers to improve knowledge and management about common under-five childhood infections with respect to behaviour change.

**Methods:**

We searched six databases between October to December 2019 using subject heading (Mesh) and free text terms in title or abstract in US English. We included multiple study types of children under-five or their caregivers who have been counselled, educated, or provided any health care service by CHWs for any common paediatric infectious diseases using mHealth. We excluded articles published prior to 1990 and those including mHealth technology not coming under the WHO definition. A data extraction sheet was developed and titles, abstracts, and selected full text were reviewed by two reviewers. Quality assessment was done using JBI tools.

**Results:**

We included 23 articles involving around 300 000 individuals with eight types of study designs. 20 studies were conducted in Africa, two in Asia, and one in Latin America mainly on pneumonia or respiratory tract infections followed by malaria and diarrhoea in children. The most common types of Health approaches were mobile applications for decision support, text message reminders and use of electronic health record systems. None of the studies employed the use of any behaviour change model or any theoretical framework for selection of models in their studies.

**Conclusions:**

Coupling mhealth with CHWs has the potential to benefit communities in improving management of illnesses in children under-five. High quality evidence on impact of such interventions on behaviour is relatively sparse and further studies should be conducted using theoretically informed behaviour change frameworks/models.

**Registration:**

PROPSERO Registration number: CRD42018117679

Despite substantial progress made under the Millennium Development Goals and the transition to Sustainable Development Goals (SDGs), global inequalities are still evident in child health. Compared with high income countries(HICs), children of the low- and middle-income countries (LMICs) are more at risk of dying due to infections [1,2]. 1055 per 100 000 children and adolescents are dying in developing countries with 6.89 million young children losing their lives of 7.7 million children and adolescents globally [3]. These deaths are attributed to several deadly yet preventable childhood infections [4]. The most noteworthy of these are pneumonia, diarrhoea, malaria, measles, typhoid, tuberculosis, hepatitis, and dengue [5,6]. Predisposing factors contributing to high incidence of these conditions include lack of education/health literacy or knowledge among caregivers, gender discrimination, religious factors, demographic and economic barriers [7,8]. These result in delays in health care seeking which is compounded by limited public health care facilities particularly in remote areas [9].

Alongside concern about the incidence of childhood infectious diseases, there has been a renewed interest in involving community health workers (CHWs) in national community health programs [10]. CHW’s are literate individuals, usually women, residing within the local rural communities and fulfilling specified eligibility criteria (eg, at least eight years of education, possession of a middle school pass, local residency, preferably married, and at least 18 years of age) hired as volunteers or against incentives (monetary or in-kind) to serve as the “focal point of care” for their communities [11,12]. According to the World Health Organization (WHO), they are trained for a shorter period of time (a few weeks to a few months) as compared to professional health care workers and although they are supported by the health system they are not a part of its organisations [13]. CHWs have been playing an important role in promoting healthy behaviours and extending the reach of the health system by acting as a bridge between the community and the system [14] through provision of health education, family planning and basic curative care for childhood illnesses [15]. However, increasingly CHWs have been asked to broaden their remits such as their involvement in community based Integrated Management of Childhood illnesses (IMCI), supporting the expansion of their involvement in curative practices [16]. This has led to complaints of unmanageable workloads [17,18]. Therefore, there is a need to develop approaches that may facilitate a reduction of this workload whilst maintaining adequate health care services/education to the communities and continuing to contribute towards reduction of infectious disease burden.

One such approach is the use of mobile technology-based health care solutions (mHealth)by CHWs. mHealth refers to the use of wireless, portable information and communication technologies (ICT) including the use of cellular phones, smart phones, personal digital assistants, tablets, or laptops to support health and health care delivery [17-19]. Typically, text, voice or video messages or various applications for public health interventions are used to increase access to care or provide information to induce health behaviour change.

With increasing penetration of mobile networks to the remotest of locations in low- and middle-income countries (LMICs), mHealth has opened new opportunities for accessible, affordable, and effective health care through CHWs [18] and is gaining momentum [12-21]. In Africa, for example, mHealth by CHWs has been used to report adverse events in intensive Multiple Drug Resistant-Tuberculosis (MDR-TB) therapy [19]. Similarly, CHWs in Uganda and Kenya have used mHealth in Acquired Immunodeficiency Syndrome (AIDS) care through text messaging [20-23]. Another study in Argentina showed the benefit of CHWs using a customised mHealth application to calculate patients’ cardiovascular risk [24]. Thus, mHealth has started to attract more attention in research with an increasing number of studies determining appropriate design of mHealth based interventions for community and health care professionals, their impacts on the outcomes of care, and barriers and enablers to scaling up [25].

For interventions aimed at inducing behaviour change (specific behavioural patterns through a ‘coordinated set of activities’), a number of models have been used [26]. The Health Belief Model (HBP) focuses on the desire to prevent an illness and the belief that a specific health related action will prevent or cure the illness. The Theory of Planned Behaviour (TPB) predicts a person’s intention to engage in a behaviour at a specific place and time thus depending on both motivation and ability. Diffusion of Innovation (DOI) Theory explains how, over time, an idea gains momentum and spreads within a specific population or social system, the end result being the individuals or social system adopting that behaviour. The Social Cognitive Theory (SCT) considers an individual’s past experiences which shape whether a person will engage in a specific behaviour and what are the reasons why that person engages in that particular behaviour. The Trans Theoretical Model (TTM)works on the assumption that behaviours are not changed quickly and decisively by people. Instead the behaviour change occurs continuously through a cyclical process. The Social Norms Theory (SNT) tries to understand influences such as the environment and interpersonal influences (peers) for behaviour change, which can be more effective than focusing on an individual to change behaviour [27]. For choosing the appropriate model, there are multiple frameworks which have been used one of which is the Behaviour Change Wheel (BCW) which recognises that the target behaviour can in arise from combinations of any of the components of the behaviour system (capability, motivation and opportunity) [26].

There is limited evidence on review of available literature on various mobile health approaches used by CHWs to improve management of children under five by caregivers especially with respect to inducing behaviour change. There has been one systematic review which has focused on use of mHealth technology by CHWs to identify “opportunities and challenges for strengthening health systems in resource-constrained settings”. However, it has not focused on management of under five children in particular [28]. We, therefore, appraised the available literature on the effectiveness of various CHW based mHealth approaches for caregivers of children to improve knowledge and/or management of common childhood infections (under five children in particular) with respect to behaviour change.

## METHODOLOGY

### Registration

The protocol was registered to PROSPERO (Registration number CRD42018117679). Ethical approval was obtained from local ethical board of International Research Force, Pakistan.

### Search strategy

#### Search databases and search terms

In order to finalize the search strategy and search terms a brief literature scoping activity in PubMed, Embase and Medline was conducted initially to explore relevant keywords and common study types. Thereafter, we conducted a systematic literature search on six databases: MEDLINE(Ovid), EMBASE(Ovid), CINAHL, PsycINFO, AMED(Ovid) and Global Health from October to December 2019. Searches were conducted in each database using both subject heading (Mesh) where available and free text terms included in title or abstract. Appendix S1 in the **Online Supplementary Document** provides a complete list of all search terms and Appendix S2 in the **Online Supplementary Document** a sample of one electronic search made in a database (Global Health). Once all the databases were searched and articles were extracted, duplicates were identified and removed using EndNote which was followed by a manual exercise for verification which involved checking the excel sheet with any redundancy. If different data or information was presented in more than one publication describing the same study, all were included.

#### Eligibility criteria

There was no restriction on geographical location and study setting and the searches were run from 1990 onwards as the first mHealth technology interventions started in the early 1990s [29]. Studies were excluded if the intervention did not fall under the WHO definition of mHealth [30], and if the study did not focus on use of mHealth by community health workers for childhood illnesses. Additionally, case studies, editorials, letter to editors, trial protocols, systematic reviews, opinion, or expert articles were also excluded. Conference abstracts were only considered if published in a peer-reviewed journal. We used the PICOS (Population, Intervention, Comparison, Outcome and Study Design) framework [31] to develop our eligibility criteria for the systematic review.

#### Population

Literature was considered for inclusion if the research included results pertaining to children under five or caregivers of children under five who have been counselled, educated, or provided with any health care service by CHWs for any common paediatric infectious disease. These paediatric infections included acute respiratory infections (pneumonia), diarrhoea, malaria, measles, typhoid, tuberculosis, hepatitis, and dengue. This age group has been selected in particular as under five childhood mortality is more than any other age group [32].

#### Intervention

We included literature that focused on the use of mHealth by CHWs. We used the definition of mHealth as specified by Global Observatory for eHealth of WHO which is *“medical and public health practice supported by mobile devices, such as mobile phones, patient monitoring devices, personal digital assistants (PDAs), and other wireless devices”* [29]. This included mHealth interventions that involve a range of delivery modes such as voice calling and text messaging via Short Message Service (SMS). It also included applicationson public health messaging, behaviour change communications, and remote care provision. Also included were applications designed to enable health workers to provide better care to patients through decision support tools for informing screening or intervention decisions, workflow planning, and clinical documentation. Additionally, global positioning system (GPS) tools for patient tracking and portable point-of-care testing devices able to transmit data via mobile phone were also included.

#### Outcome

Outcomes included any change in knowledge, perception, awareness, insight, behaviour, or familiarity among caregivers of children and/or community health workers about common childhood infections. Additionally, we sought any impact on hospitalisation eg, number of days in hospital, improved efficiency of the CHWs in managing workload, and improved clinical outcomes (childhood morbidity and mortality).

#### Study designs

The documents included were randomised controlled trials (RCTs), pilot/feasibility studies, quasi-experimental studies, cohort studies, qualitative studies, cross-sectional studies, and project evaluations which focused on assessing the impact of using mobile health technology by CHW for infectious diseases in children under five.

### Quality assessment

The Joanna Briggs Institute (JBI) Critical appraisal tools were used to assess risk of bias. The JBI tools which were used for appraisal included those for RCTs, qualitative studies, quasi-experimental studies, cross-sectional studies, cohort studies and economic evaluations [33]. HM and SN conducted appraisal of all included studies and scored them independently. Results were presented as overall mean quality score, while we defined it as, summing mean score of both appraisers and dividing it by the number of appraisers. However, mean score was calculated by dividing sum of individual item score with total number of quality items. Individual quality item was scored either as 1 (ie, present) or 0 (ie, absent)

### Data synthesis and analysis

Titles, abstracts, and selected full text were reviewed by two reviewers (HM and SN). A data extraction sheet was used to extract all relevant information which included the title, author(s), year of publication, country, health care setting, aims and objectives, study design, sample size, target users, type of infection, type of mHealth approach, duration, key findings, strengths and limitations. Data extraction was done by one reviewer and reviewed by the other. We intended to conduct data synthesis if suitable comparable RCTs were found, however, due to heterogeneity in their intervention and outcomes, descriptive analysis of the attained data was conducted.

## RESULTS

**Figure 1:**
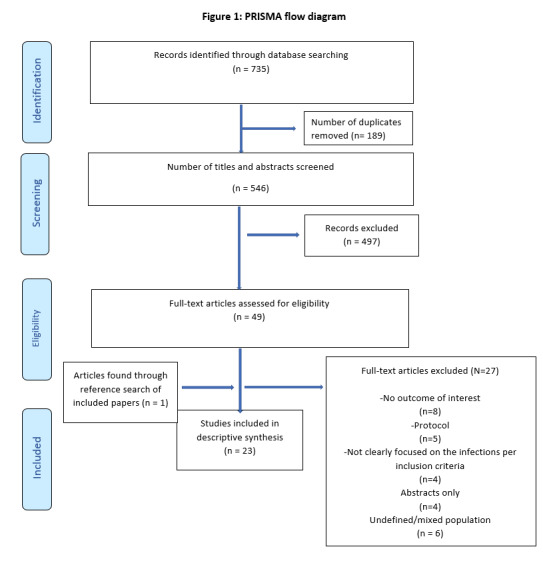
PRISMA flow diagram.

A total of 736 articles were obtained from all the databases. 189 duplicates were removed leaving546 articles for title and abstracts review. A list of 49 articles were then extracted from these based-on inclusion criteria and then on full text assessment and consensus between the reviewers, 23 articles were selected for final stage. The PRISMA flow in **Figure 1** indicates the process of selection.

### Study types

The 23 articles reviewed described eight type of study designs; six RCTs [34-39], six quasi experimental studies [40-45], six qualitative studies [44,46-49], one case study [50], one cohort study [51], and one cost evaluation study [36] and one mixed method study [52].

### Study settings

Most articles reported on projects in economically developing countries particularly Africa, with several focused-on Asia, and a few in Latin America as indicated in **Table 1**. Among the countries reported, most of the studies were conducted in Kenya (n = 5), followed by Uganda (n = 4), Malawi (n = 3) and Ghana (n = 2) with the rest of the studies in other countries.

**Table 1:** Summary of findings

Sr.	Author, year, country, target group, infection, and sample size	Methodology	Key findings	Behaviour change observed	Limitations
**Randomized control trial**					
**1.**	Zurovac 2011 [36], Kenya, 119 health workers and 624 children under five, Malaria	Text message reminders in the form of ten tasks about paediatric malaria case management (treatment, dispensing and counselling) based on Kenyan national malaria guidelines were sent to health workers doing outpatient consultation for 6 mo. Three health facility surveys were conducted: baseline; one at 6 months of intervention, and one 6 months after the intervention. Primary outcome was a composite indicator for correct artemether-lumefantrine management among recruited children based on accomplishment of the 10 specified tasks.	In the intention-to-treat analysis, correct artemether-lumefantrine management was improved by 23 · 7% (95% CI 9.0-33.7, *P* = 0.0007) immediately after the intervention and by 24 · 5% (11.6-35.7, *P* = 0.0001) 6 months after the intervention. Improvements of similar eﬀect size were also observed when the performance indicator was all four treatment tasks and at least ﬁve of six dispensing and counselling tasks; 21.4% (improvement soon after the intervention 95% CI 9.0-33.7, *P* = 0.0007) and 23.7 improvement 6 months post intervention % (11.6-35.7, *P* = 0.0001). Eﬀect sizes on per protocol analysis were larger as correct artemether-lumefantrine management improved by 31 · 7% (95% CI 15.6-47.8) immediately after intervention and 28.6% (12.7-44.6) 6 months after the intervention.	Improved carer performance on doing the ten tasks by 10 · 3%(4.0-16 · 6, *P* = 0 · 0013) immediately after the intervention and 11 · 3% (5.1-17.6, *P* = 0 · 0004) 6 months after the intervention ended.	Messages were not sent in native or national language of Kenya. Intervention did not address need to assess children who would test negative for malaria.
**2.**	Donovan 2018 [35], Uganda, 129 CHWs, Pneumonia	Mobile tablets were uploaded with videos on pneumonia, its recognition, its treatment and prevention for intervention group which received one and half day training on the standard iCCM guidelines followed by tablet-based video training; pre and post training test. Primary outcome was knowledge acquisition and retention as determined by changes in MCQ scores.	Independent samples *t* test showed mean improvement in MCQ scores to 2.6 (SD ± 3.1) in the control group (10.8%) and 3.2 (SD ± 2.3) in the intervention group (13.3%), but the difference between both the groups with respect to improvement (mean 0.6; 2.5%) was not statistically significant (*t* = 1.15, *P* = 0.254). Years of education was positively associated with MCQ change scores, with a beta coefficient of 0.33 (*P* = 0.02) though multiple regression. Additionally, Pearson correlation coefficient between pre-training scores and change scores of MCQs was -46 (*P* < 0.001).	-	Short duration of one month for the pilot. Use of non-validated assessment tool.
**3.**	Li Chen 2016 [39], China, village doctors, caregivers of children; 7 participants from each cluster of 18 pairs of clusters. Hepatitis B and Measles	Intervention group village doctors used mobile phones with the EPI app to manage child vaccination coupled with text message dissemination to alert caregivers about upcoming vaccinations. Two cross-sectional household surveys were conducted at baseline and end-line. Face-to-face in-depth interviews with village doctors were also conducted at the end of the study.	Increase in full vaccination from baseline to end-line in both the intervention and control group ie, from 67% (95% CI:58%-75%) to 84% (95% CI:76%-90%), *P* = 0.028 in intervention group and from 71% [95% CI = 62%-79%] to 82% [95% CI:74%-88%], *P* = 0.014 in control group. Higher increase was observed in intervention then in control group from baseline to end-line (17% vs 10%), but this was not statistically significant (*P* = 0.164). Village doctors reported that their management of child vaccination saved time. The education module in the app let them learn vaccination related key knowledge and skills conveniently. However, they did indicate it was hard to manage migrated children.	Increased follow up by caregivers for timely vaccination.	Short project duration.
**4.**	Zakus 2019 [34], Southwest Niger, 31 CHW, 252 children between 2-59 mo. Diarrhoea, malaria, and pneumonia.	Both control and intervention group CHWs (RComs), were trained on iCCM; intervention group had additional training on applying iCCM through a mobile application which also contained module on control of drugs and supplies. Each RCom was visited by a trained clinician and an assistant per district to assess QoC and levels of motivation and retention.	A 3.4% higher QoC score was shown by mHealth equipped RComs with a mean difference of 0.83 points. These RComs were more likely to inquire about danger signs with convulsions at 69.7% vs 50.4%, *P* < 0.001, incapacity to eat or drink at 79.2% vs 59.4%, *P* < 0.001, vomiting at 81.4% vs 69.9%, *P* < 0.01), and lethargy or unconsciousness at 92.4% vs 84.8%, *P* < 0.01. A QoC score of more than 80% (25 out of 31) was observed among 83%RComs of the intervention group had a as opposed to only 67% RComs in the control group. Correct referrals were 85% in intervention as compared to 29% in control. no statistically significant differences in motivation, retention, and supervision.	-	Initial and subsequent training on using the smartphone could have been better, along with closer and more specific supervision to use the technology to maximum effect to leverage effectiveness of the iCCM program.
**5.**	Davis 2019 [37], Uganda, 387 household contacts; children from <5 y to 14 y, >15 y without HIV, PLHW. Tuberculosis	CHWs for both intervention and standard care arms were trained to provide home sputum collection and HIV counselling and testing services according to Uganda National Guidelines. Intervention group CHWs entered all information in a survey application, which applied an algorithm to characterise each contact’s need for further evaluation. Sputum testing results and/or follow-up instructions were returned by automated SMS texts. Primary outcome: Completion of a full TB evaluation within 14 d of treatment.	CHWs identified 190/471 (40%) intervention and213/448 (48%) standard care contacts requiring TB evaluation. CHWs obtained sputum from 35/91 (39%) of sputum-eligible contacts and text messages were sent to 95/190 (50%) of contacts in the intervention arm. In both the intervention and standard care arms, completion of TB evaluation at 14 d was 14% and 15% respectively with a difference of -1% at 95% CI (-9% to 7%, *P* = 0.81). However, yields of confirmed TB diagnosis (1.5% in intervention and 1.1% in standard care, *P* = 0.62) and new HIV diagnosis (2.0% in intervention vs 1.8% in standard care, *P* = 0.90) were similar.	-	Non review of clinic registers. Incomplete delivery of intervention, difficulties in collecting sputum.
**6.**	Talisuna 2017 [38], Uganda, 1677 children <5years and their caregivers. Malaria	It was an open-label, randomized, controlled trial assessing effects of SMS reminders on patients’ adherence to AL. Participants in intervention and control group were randomized into 3 categories: category 1: caregivers were visited at home on day 1 to measure adherence of the second and the third dose of AL; category 2: caregivers were visited at home on day 2 to measure adherence of fourth and fifth AL doses and, category 3: caregivers were visited at home on day 3 to measure adherence to full treatment. Caregivers in the intervention arm were also sent automated SMS reminders on treatment adherence. Primary outcomes were: (a) the proportion of patients adhering to complete AL course and, (b) the proportion of patients’ returning to the facility on day 3.	Randomization of all enrolled children: 849 (50.6%) into control group and 829 (49.4%) into intervention group. Of the 562 children visited at home on day 3 to measure full treatment adherence, all doses were given to 97.6% (282/289) of children in the control and 97.8% (267/273) in the intervention group (OR = 1.10; 95% CI = 0.37-3.33; *P* = 0.860). On assessment of correct timing of taking each dose, 72.3% (209/289) were adherent in the control and 69.2% (189/273) in the intervention group (OR = 0.82; 95% CI = 0.56-1.19; *P* = 0.302). The odds of children returning to the facilities on day 3 and 28 within the intervention group were also increased by sending SMS reminders; day 3 (81.4 in vs 74.0%; OR = 1.55; 95% CI = 1.15-2.08; *P* = 0.004), 28 (63.4 vs 52.5%; OR = 1.58; 95% CI = 1.30-1.92; *P* < 0.001).	Improved adherence to treatment by caregivers and improved follow up visit in health care facility	-
**Quasi-experimental:**					
**7.**	Xeuatvongsa 2016 [45], Lao People Democratic Republic, health care workers, health care volunteers, 9319 children up to age of one year. Hepatitis B	6-mo Intervention; district-level non-random assignment. Intervention health workers received a one-day training on using phones in case of imminent delivery, mother/baby with danger signs, birth notification, PNC services HCWs provision, and administration of HepB vaccine. Comparison district workers did not have phone related training. Study also included a household evaluation survey on difference in the change of HepB-BD coverage between intervention and comparison districts.	The median difference in village level Hep-B vaccination coverage was 57% (interquartile range [IQR] 32%-88%, *P* < 0.0001) in intervention districts, compared with 20% (IQR 0%-50%, *P* < 0.0001) in comparison districts. Intervention districts showed more improvement than in control districts (*P* = 0.0009).	Caregivers responded to referrals by health workers for Hep B vaccination increasing coverage from 20% to 57%.	Results not generalizable, several selected villages only had children from one age group, making comparison impossible, some villages had 100% coverage at baseline and therefore no further improvement could be expected.
**8.**	Tumusiime 2014 [43], Uganda, CHWs, children under age of five, caregivers of children, local leaders & government health officials. Diarrhoea, acute respiratory infection (ARI), pneumonia	9-mo study on development of a mobile application for timely diagnoses, recognition of danger signs, communication about referrals and initiation treatment through a specially designed mobile application used by CHWs. The entire implementation process with development of mobile algorithms, completion of an environmental scan with training of 96 CHWs, took place between July 2011 and March 2012	The project was completed in 7 phases. Phase 1 included development of interface and its testing, Phase 2 usability testing was accomplished, Phase 3 included the environmental scan, Phase 4 reported on phase 3, Phase 5 included the procurement process, Phase 6 included uploading the phones with application and phase 7 was development of training manual. Upon training CHWs demonstrated the capacity to contribute significantly to the timely gathering of data on incidence, referral, and treatment of childhood illness. The quality of the information obtained using the mobile phones surpassed that obtained from conventional paper records. Additionally, retrieval of data along with analysis from mobile phone records was greatly facilitated.	Improved care seeking by caregivers upon referrals through CHWs using the mobile application.	Recruitment of CHWs and the demonstration of the controlled intervention trial lacked detail. The study was not able to prove long term benefits of using mobile phones in rural Uganda and similar settings.
**9.**	Kabakyenga 2016 [44], South Western Uganda,196 CHWs, 1529 children under age of five, caregivers of children. Malaria, pneumonia & diarrhoea.	An observational study in five parishes (47 villages) served by CHWs well versed in iCCM with supplemental training in mobile phone use. Impact was assessed by quantitative measures and qualitative evaluation through household surveys, key informant interviews and focus group discussions.	CHWs supported by mobile phones correctly treated 97.1% of fever cases, 88.2% of pneumonia cases and 92.4% of diarrhoea cases. Whereas trained CHWs without mobile phone appropriately treated fever in 93.6% cases, pneumonia in 92.3% and diarrhoea in 90.4%. However, significant improvements in clinical outcomes when compared between mobile phone and non-mobile phone supported CHWs were unproven in this demonstration. Qualitative evaluation showed improvements in treatment planning, supply and logistical management, and efficiency.	**-**	Small sample size and limited observation period. Lack of information on development of the mobile phone tool and mitigation of any challenges encountered.
**10.**	Ndiaye 2018 [40], Senegal, 44 682 children, caregivers and 91 health workers, Malaria	Two strategies to improve reporting of acute emergencies (AEs) during SMC campaigns were evaluated as compared to the national spontaneous reporting system. Health posts were allocated into three arms: Safety monitoring using the national system of spontaneous reporting through the national reporting form (the national system), completed by physicians or nurses at health facilities using mobile phones (enhanced spontaneous reporting), completed by nurses at health posts and by CHWs with active follow-up of children at home after to inquire about AEs and collect record on a symptom card (active surveillance).	Rate ratios were used to compare rates between surveillance methods, age groups, and calendar months, estimated using Poisson regression1145 events were reported over 3 months with a rate of 30.6 (95% CI = 28.8-32.4) per 1000 children treated per month, compared to 1.65 (95% CI = 1.27-2.15) per 1000 per month in health posts using national system. Enhanced reporting with CHWs using mobile phones also increased reporting by 18-fold (rate ratio 18.5, 95% CI 8.65-39.7). The most commonly reported symptoms were fever, vomiting and abdominal pain. No serious adverse drug reactions were detected despite increased surveillance.	**-**	The lack of suitable controls to establish the rate of symptoms in children who did not receive SMC. There might have been reporting bias, both by caregivers and by CHWs. Caregivers because they might have not reported on symptoms not listed on the symptom card, and CHWs because their training emphasized on the known side effects of SMC drugs.
**11.**	Finette 2019 [42], Burkina Faso, Ecuador, and Bangladesh, 861 children 2-60 months of age and 49 CHWs. Pneumonia, diarrhoea, malaria.	Study describes development and initial validation testing of an mHealth platform, MEDSINC designed for health workers to perform clinical risk assessments of children. Clinical assessments made by CHWs through MEDSINC were correlated blindly and independently with those madeby22 local health care professionals (LHPs).	Results showed an 84% and 99% correlation between CHW generated assessments and those conducted by HCPs. Triage recommendation distributions of MEDSINC were highly correlated with those of Local health care providers whereas usability and feasibility responses were collectively positive for ease of use, learning, and job performance.	-	Less accurate assessment of specificity and sensitivity.
**12.**	Boyce 2019 [41], Malawi, 799 HSAs in the trial and 47 KIIs with stakeholders representing all levels of the iCCM implementation System and children 2-59 months of age. Pneumonia, diarrhoea, malaria	This study compared the use of an iCCM enabled mobile application by CHW (HSAs) to paper-based management tools. This was further supplemented by conduction of 47 key informant interviews about the perceptions of HSAs on Quality of Care (QoC) and sustainability of the iCCM based mobile application.	Mobile phone enabled HSAs assessed sick children based on iCCM guidelines more often than HSAs using paper-based tools for cough (adjusted proportion, 98% vs 91%; *P* < 0.01) and five physical danger signs including chest in-drawing, alertness, palmar pallor, malnourishment, and oedema (80% vs 62%; *P* < 0.01), but not for and diarrhoea (94% vs 87%; *P* = 0.03). 81% of mobile based HSAs correctly classified ill children based on danger signs as compared to 58% of HSAs using paper-based tools (*P* < 0.01). No differences existed for their treatments (*P* = 0.27). Interview respondents stated that using mobile application ensures protocol adherence. Barriers to consistent and wide use included hardware problems and limited resources.		The study was not part of the initial program design, researchers could not randomize the intervention nor have a baseline assessment. They were, therefore, unable to determine if any differences existed between the groups prior to the implementation of the mobile application.
**13.**	Ismail 2017 [50], Kenya, 9 months to 14-y-old children; 53 277 villages in the 46 counties. Measles and rubella	In this study use of a mobile phone application was assessed for national level planning and implementation of a measles rubella (MR) campaign in Kenya. Data collection was done using 7 data collection forms (village forms)	Real time data was received from 46 of 47 counties. The microplanning process was done within a very short time of 4 weeks, compared to the 2013 polio micro plans which took more than one year to be submitted to the national level. More than 3 million children who were not captured in the national plan were captured by the micro-plans. 98% had mapped all the places where the target age children could be found. However, the uploading of the data by the sub county teams were not getting as fast as planned with incomplete drafts in the system leading to clogging.	-	Unclear background and introduction and methodology of data collection.
**14.**	Althaus 2017 [52], Tanzania, 150 children aged 2-59 mo, health workers. Malaria, pneumonia, UTI, dysentery, diarrhoea, typhoid fever.	The study assessed the impact of an electronic Algorithm for management of childhood diseases on Healthcare worker performance and antimicrobial prescription. Nine primary health care facilities (HFs) were randomized into three arms: 1) paper algorithm, 2) smartphone based electronic algorithm and 3) control. Main outcomes: Proportion of children checked for danger signs Proportion of children given antibiotics.	Use of electronic tool by CHWs vs paper led to a significant increase in children checked for danger signs (41% vs 74%, *P* = 0.04). In control arm, dangers signs were checked in only 3% of the children (range: 2%-4% among three HFs), In the paper arm, danger signs were checked in 41% of the children (range: 16%-71% among three HFs, aRR as compared with control arm, 95% CI: 14.4, 95% CI=3.4-69.7,), whereas in the electronic arm, danger signs were checked in 74% of the children (range: 63%-94%, 30.9, 95% CI=9.2-120.2).Two-thirds of the children had their main symptoms checked in the control arm (77%, range: 64%-91%) and the paper arm (75%, range: 68%-82%, 1.0 [0.8-1.2]), whereas in the electronic arm, almost all the children had their symptoms checked (99%, range: 98%-100%, 1.3, 95% CI=1.2-1.3. Additionally, the proportion of children with CHWs’ disease classifications matching that of the experts was low in both control (34%, range in the three HFs: 22%-56%) and paper arms (39%, range: 35%-42%, 1.1, 95% CI=0.7-1.9) but slightly higher in the electronic one (53%, range: 47%-59%, 1.6, 95% CI=1.0-2.5). Similarly, the proportion of children prescribed antibiotics was much lower in the interventions than in the control arm (70%, range 60%-85% in the control; 26%, range 14%-37%, 0.4, 95% CI=0.2-0.6) in the paper; and 25%, range: 17%-33%, 0.3, 95% CI=0.2-0.5 in the electronic arm.	-	The small number of HFs involved, their disparities in size, and the relatively small number of consultations observed limits the power of the analysis.
**Qualitative study:**					
15.	Jones 2018 [48], Kenya, 34 caregivers of children. Malaria	The study (through FGDs of caregivers from both intervention and control arms) explored participants’ experiences in an RCT trial on effects of text message reminders on paediatric adherence to artemether-lumefantrine(AL) and identification of factors that contribute to high adherence rates.	Intervention-arm participants reported that text messages were effective dosing reminders. Caregivers from both arms mentioned that in depth instructions played an important role in treatment adherence. They also mentioned that among the contributing factors to high quality care and adherence to dosing instructions, respectful and personalized treatment of caregivers from trial CHWs was forefront.	-	Gap between trial end and FGD of was long to have affected the memory.
16.	Ginsburg 2016 [46], Ghana, 71 respondents; District Health administrators, health care assistants, community health officers (CHOs), community health nurses (CHNs) and caregivers of 2 mo- and 5 y-old children. Pneumonia.	A design-stage qualitative pilot study was conducted to assess feasibility, usability, and acceptability of mPneumonia (mobile application to diagnose, classify and manage pneumonia) in six health centers and five community-based health planning and services centers.	Health administrators reported app would be useful if approved by national and regional decision makers. HCPs felt using the app would improve accurate patient care. They stated that the application was easy to use and provided the health workers confidence in diagnosis and treatment of children. Major challenges of application were electricity requirements for charging and additional time required to complete the application. Some caregivers saw the app as a sign of modernity, increasing their trust in the care provided to their children. A few of the caregivers were slightly hesitant and/or confused regarding the new technology.	-	Influence on time spent per patient due to additional assessment of pneumonia cases per standard of care.
17.	Bessat 2019 [47], Burkina Faso, 21 health workers. Common childhood illnesses	This study was conducted in the frame of a large-scale implementation of an e-IMCI tool developed. 12 in-depth interviews and 2 focus-groups were conducted from health workers of 10 primary care facilities. Themes were identified through qualitative data analysis software.	Users showed a high level of satisfaction although one of the major inconveniences perceived was slowness of the tablet. Several common illnesses were identified as missing in the algorithm along with guidance for fever. Only five users stated that antibiotics had no action on viral diseases. The tool was perceived to be improving patient management and rational use of antibiotics. Positive changes in health facility organisation were also reported, such as task shifting and improved triage.	-	Influence of researcher on research and vice versa were not addressed. Results may not be generalizable to urban settings.
18.	Ide 2019 [41], Malawi, 17 HSAs and 28 caregivers. Malaria, diarrhoea, and pneumonia	This study was conducted in the frame of a large-scale implementation on use of an mhealth tool on iCCM. Data was collected through semi-structured interviews with HSAs and caregivers. Deductive and inductive approaches were used during data analysis.	Nearly all HSAs preferred the App over routine paper based CCM. Most of the HSAs stated that the application was less prone to errors and therefore more reliable, facilitating more accurate diagnoses and treatment of children. It also led to enhanced professional confidence and respect within the community. A few also mentioned that they did not trust the results blindly. Caregiver reactions to the App’s validity was mixed but leaned towards favourable. Many HSAs also welcomed the mobile technology as the way of the future and also felt it was acceptable within their community. Usability features included faster provision of care, portability, improved durability, and more efficient and easier monthly reporting to the District Health Officer. Inadequate mobile network coverage or electricity shortages were the main challenges.	-	Influence of researcher on research and vice versa were not addressed.
19.	Ginsburg 2015 [46], Ghana, 7 HCPs	This study was a design-stage usability field test of a mobile application (mPneumonia) with the aim of developing a user-friendly diagnostic and management aid for childhood pneumonia that would improve diagnostic accuracy and facilitate adherence by health care providers to established guidelines in low-resource settings	All HCPs expressed a desire and willingness to use the application. They, however, felt that nominal training and adequate technical support would be required for first-time users. Overall, all HCPs preferred to use mPneumonia over the paper-based tools.	-	Small sample size. No statement locating researcher culturally or theoretically. Influence of researcher on research and vice versa were not addressed.
20.	Svege 2018 [39], Malawi, 375 caregivers. Malaria	Study was conducted to determine which strategy (standard care vs text message reminder based)is best suited for large-scale and long-time implementation of post-discharge malaria chemoprevention (PMC) in areas of high malaria transmission.30 in-depth interviews and 5 focus group discussions were conducted with caregivers of children who recently completed the last treatment course in a randomised placebo-controlled trial using text messages on PMC.	Lack of money for travel expenses was identified as one of the main hurdles in the facility-based study arms whereas a major strength was increased follow-up care and continuous contact with health personnel. Most of the respondents in facility-based study arms were in favour of the drug delivery through a community-based approach. Informants preferred text message reminders sent directly to their phones rather than waiting on these visits and described text messages as a “quick” and “easy” way of conveying reminders directly to caregivers. Barriers included the challenge of phone usage, lack of electricity, inadequate charging services and network problems. Although caregivers majorly characterised HSAs as helpful and generous, there were some who called them lazy and negligent. The majority of respondents ranked text messages as their preferred method across all study arms.	Increased follow up care and continuing contact with health care personnel along with commitment to comply with treatment guidelines.	Reduced male participation. No statement locating researcher culturally or theoretically. Influence of researcher on research and vice versa were not addressed.
**Cost evaluation study:**					
21.	Zurovac 2012 [53], Kenya,119 health workers for scenario 1, 20 000 for scenario 3. 153 379 children for scenario 1 and 2. 3 million children for scenario three.	Study describes costs and cost-effectiveness under three implementation scenarios: (1) as implemented under study conditions in study areas; (2) if the intervention was routinely implemented by the Ministry of Health in same areas; and (3) if the intervention was nationally scaled up.	Under the study conditions, various costs of the intervention were found to be USD19 342 whereby 45% was for developing and pretesting of text-messages, 12% for developing text-message dissemination system, 29% for collecting health workers’ phone numbers, and 13% for sending text-messages and monitoring of the system. It was estimated that if this were implemented by the MoH, the costs would be 28% lower (USD13,920) attributed to lower costs of collecting health workers’ numbers. National scale up cost would be USD97 350 with majority costs (66%) for disseminating text-messages. The cost per additional child correctly managed was USD0.50 under study conditions, USD$0.36 if implemented by the MoH, and USD0.03 if implemented nationally.	-	Did not test frequency and duration of reminders. Did not focus on duration beyond 6 months of intervention.
**Mixed methods study:**					
22.	Richards 2016 [52], Ethiopia, 57 respondents; policy makers, health care providers: health-extension-workers, health centre heads, district health officers, Zonal Health Department (ZHD) representatives and Regional Health Bureau (RHB) HMIS Officers. Tuberculosis	The study assessed feasibility of using eHealth by female health extension workers (HEWs) within their core duties on tuberculosis, maternal child health, and gender equity. Mixed method baseline data collection was undertaken through quantitative questionnaires (n = 57) and purposively sampled qualitative semi-structured interviews (n = 10) and focus group discussions (n = 3).	67% of the 12 DHOs, 81% of the 27 HCHs and all the 18 HEWs did not know what eHealth is. Mobile phone communication was valued and used by HEWs for enabling clients to access health facilities, coordinating care, sharing information with colleagues and offices, and obtaining resources. In some cases, network non availability and difficulty charging were an issue. Ability to access information and organize it was perceived to be the most beneficial element of HMIS by most HEWs. Delay of 3-7 d of receipt of HMIS reports and English language as a barrier was highlighted in traditional system. Effective health care delivery, monitoring and evaluation of their performance in delivering the 16 health packages was reported as their main role by all HEWs. Thus, the Health Management Information System (HMIS) was seen as important by all participants, but with challenges of information quality, accuracy, reliability, and timeliness.	-	Inability to assess the distance and frequency of travel of HEWs to access the Internet, and lack of quantitative data to verify the data inconsistencies.
**Cohort study:**					
23	Meyers 2016 [51], Nepal, 16 Community health workers, 2710 cases of Diarrhoea & 373 Acute Respiratory Infections	The study aimed to evaluate if community-based surveillance systems can capture temporal trends in acute respiratory infections and diarrhoea. It compared the infection rates from community (through mobile phone-based data collection by CHWs) and hospital and assigned three levels of disease activity (low, medium, and high) to each week for 12 mo.	CHWs reported 373 cases of ARI and 2710 cases of diarrhoea. Using a square root transformation of each community and hospital-based rate, the authors categorized the transformed community health rates by tertiles: low, medium, and high. Results showed that for diarrhoea, there were significant differences between low vs high (*P* = 0.001) and medium vs high (*P* = 0.04) tertiles in post-hoc comparisons between hospital and CHW rates whereas for ARI, the only significant difference was between low vs high (*P* = 0.01).	-	Severity of illness was not catered as this might have led to discrepancy as the hospitals usually attend more severe cases and less severe usually do not reach the facilities. Comparator of hospital data are not gold standard.

CI – confidence interval, iCCM – integrated community case management, SD – standard deviation, MCQ – multiple choice question, EP I- expanded program of immunization, CHW – community health worker, QoC – quality of care, HIV – human immunodeficiency virus, TB – tuberculosis, SMS – short message service, PNC – prenatal care, HCWs – health care workers, Hep B – hepatitis B, ARI – acute respiratory infections, AEs – acute emergencies, HCPs – health care professionals, KII – key informant interviews, MR – measles, rubella, UTI – urinary tract infections, HFs – health care facilities, FGD – focus group discussions, AL – artemether-lumefantrine, CHO – community health officers, CHN – community health nurses, e-IMCI – electronic integrated management of childhood illnesses, PMC- post-discharge malaria chemoprevention, USD – United States dollar, ZHD – zonal health department, RHB – regional health bureau, HMIS – health management information system, DHOs – district health officers, HCHs – health care heads, HEWs – health extension workers

### Participants and illnesses

Among all the articles whereby an mHealth approach was used with/through CHWs, ten of those targeted caregivers along with children with the rest focused on children alone. The target beneficiaries numbered approximately 300 000 individuals.

Most of the articles (n = 11) focused on pneumonia or other respiratory tract infections. The next most common infections targeted were malaria (n = 10), diarrhoea (n = 5), two articles reported interventions addressing hepatitis, measles, and TB, and one each on rubella and typhoid fever. The majority of articles (n = 11) included various illnesses together.

**Table 1** provides a summary of findings of the included studies.

### Quality assessment

**Table 2** shows the quality assessment of the studies using JBI tools. Overall, evidence was of moderate quality. Among the RCTS, three of the six covered all aspects of randomization, allocation concealment, blinding, follow up and reliable outcome measurement. Most of the quasi-experimental studies could not provide clear information on measurement of both outcome and exposure along with follow up. The included qualitative studies addressed all quality parameters except for ‘researcher influence on the research’. Two studies, the cross-sectional and the cohort, did not address the majority of the quality parameters.

**Table 2:** Quality assessment

Randomized control trials																			
**Sr N**	**Study characteristics**		**Zurovac et al [**36**]**		**Donovan et al [**35**]**			**Li Chen et al [**39**]**		**Zakus et al** [34]					**Davis et al** [37]		**Talisuna et al** [38]		
1.	Randomization		Y		Y			Y		Y					Y		Y		
2.	Allocation concealment		Y		N			U		U					Y		Y		
3.	Blinding		Y (Nn- blinding of treatment providers and outcome assessors)		N			U		U					Y (Nn- blinding of treatment providers and outcome assessors)		N		
4.	Follow up complete		Y		U			Y		U					Y		Y		
5.	Reliable outcome measurement with use of appropriate analysis		Y		Y			Y		Y					Y		Y		
**Quasi-experimental studies**																			
	**Study characteristics**		**Xeuatvongsa et al [**45**]**	**Tumusiime et al [**43**]**		**Kabakyenga K et al [**44**]**					**Ndiaye et al [**40**]**			**Finette et al [**42**]**			**Boyce et al [**41**]**	**Ismail et al [**50**]**	**Althaus et al [**52**]**
1.	Clarity on cause and effect		Y	Y		Y					Y			Y			Y	Y	Y
2.	Inclusion of control		Y	N		N					N			Y			Y	N	Y
3.	Similarity of comparisons		Y	Y		U					N			Y			Y	U	Y
4.	Measurement of both outcome and exposure		U	Y		U					U			U			Y	Y	Y
5.	Complete follow up		U	U		U					Y			U			U	U	U
6.	Reliable outcome measurement with use of appropriate analysis		Y	N		N					Y			Y			Y	Y	Y
**Qualitative studies +1 mixed method (Richards et al [**52**])**																			
	**Study characteristics**	**Jones et al [**48**]**			**Ginsburg et al [**46**]**		**Bessat et al [**47**]**		**Ide et al [**41**]**				**Ginsburg et al [**51**]**			**Svege et al [**39**]**	**Richards et al [**52**]**		
1.	Congruity b/w research methodology and research question	Y			Y		Y		Y				Y			Y	Y		
2.	Congruity b/w research methodology and data collection methods	Y			Y		Y		Y				U			Y	N		
3.	Congruity b/w research methodology and data analysis	U			Y		Y		Y				Y			Y	Y		
4.	Congruity b/w research methodology and result interpretation	Y			Y		Y		Y				Y			Y	Y		
5.	Researcher influence addressed	N			Y		N		N				N			N	N		
6.	Participant voices represented	Y			Y		Y		Y				Y			Y	Y		
**Cross-sectional studies +1 mixed method**																			
	**Study characteristics**											**Richards et al [**52**]**							
1.	Clearly defined inclusion criteria											U							
2.	Detailed description of study setting and study subjects											Y							
3.	Valid and reliable measurement of exposure											U							
4.	Use of objective, standard criteria for measurement of condition											U							
5.	Identification of confounding factors											N							
6.	Reliable outcome measurement with use of appropriate analysis											U							
**Cohort studies**																			
	**Study characteristics**											**Meyers et al [**51**]**							
1.	Similarity among two groups and recruitment from same population											N							
2.	Similarity in exposure measurement											Y							
3.	Valid and reliable measurement of exposure											Y							
4.	Identification of confounding factors											U							
5.	Valid and reliable measurement of outcome											Y							
6.	Follow up complete											N							
7.	Reliable outcome measurement with use of appropriate analysis											Y							
**Cost evaluation**																			
	**Study characteristics**											**Zurovac et al [**53**]**							
1.	Well defined question											Y							
2.	Comprehensive description of alternatives											Y							
3.	Identification of all important and relevant costs and outcomes for each alternative											Y							
4.	Established clinical effectiveness											U							
5.	Accurate measurement of costs and outcomes											Y							
6.	Costs and outcomes valued credibly											Y							
7.	Costs and outcomes adjusted for differential timing											U							
8.	Incremental analysis of costs and consequences											Y							
9.	Study results include all issues of concern to users											U							
10.	Generalizable results											Y							

*Yes – present, No – absent, U – unable to identify

### mHealth technology

In the majority of the studies, the focus was the use of mHealth as an adjunct to the regular activities of the CHWs. The most common types of mobile phone approaches were use of mobile applications for decision support, text message reminders and applications for electronic health record system. However, none of these approaches used a theoretically informed behaviour change model while developing the intervention. **Table 3** summarises the various mHealth approaches across the studies.

**Table 3:** mHealth approaches

Sr. No	Studies	mHealth approach			Theoretically informed behaviour change model
		**Decision support through mobile application**	**Text message reminders**	**Electronic health records**	**Yes/No**
1.	Zurovac D et al (1&2) [36,53]	-	One-way communication of text-message reminders on paediatric malaria case-management accompanied by “motivating” quotes through an automated message delivery system.	-	No
2.	Xeuatvongsa et al [45]	-	To facilitate communication between the Volunteer health workers and health care providers to improve Hep B immunization rates	-	No
3.	Tumusiime D et al [43]	mHealth based application on integrated community case management (ICCM)	-	-	No
4.	Kabakyenga K et al [44]	Use of mobile phones augmenting integrated community case management (ICCM)	-	-	No
5.	Ndiaye et al [40]	-	-	Reporting of adverse events of chemoprovectin using mobile phones (enhanced spontaneous reporting), completed by nurses at health posts and by Community health workers.	No
6.	Finette et al [42]	Mobile application through physician-based logic to generate integrated clinical risk assessments, triage, treatment, and follow-up recommendations for common childhood illness management	-	-	No
7.	Boyce et al [41]	mHealth based application for ICCM	-	-	No
8.	Althaus et al [52]	mHealth based application for ICCM	-	-	No
9.	Jones et al [48]	-	Reminders for caregivers on adherence to malaria management guidelines	-	No
10.	Ginsburg et al [46]	Mobile Application to diagnose, classify, and manage childhood pneumonia	-	-	No
11.	Bessat et al [47]	Mobile application on treatment decision making, dosage calculation, standardization of treatment and rational use of medication for common childhood illnesses	-	-	No
12.	Ide et al [41]	mHealth based application for ICCM	-	-	No
13.	Svege et al [39]	-	Reminders for caregivers to obtain medications for malaria from health facilities through timely follow up	-	No
14.	Richards et al [52]	-	-	Health management information system on tuberculosis	No
15.	Meyers et al [51]	Decision support by tracking patients, follow up and next steps of care for common respiratory and diarrhoeal illnesses	-	Patients follow up data entry.	No
16.	Ismail et al [50]	-	-	Patient data collection through mobile application in Measles and Rubella campaign	No
17.	Donovan et al [35]	Tablets with application containing videos on detection, treatment, and prevention of pneumonia.	-	-	No
18.	Li Chen et al [39]	-	-	Mobile application with four modules: 1) making appointments; 2) recording vaccination status; 3) tracking overdue children; and 4) providing education	No
19.	Zakus et al [34]	Decision support for iCCM and management of drugs and supplies for childhood malaria, pneumonia, and diarrhoea	-	-	No
20.	Davis et al [37]	-	SMS-facilitated household TB contact investigation	-	No
21.	Talisuna et al [38]	-	SMS based reminders to caregivers of children under five to adhere to national antimalarial guidelines. Reminders were also sent for follow up visits to the health facilities.		No

mHealth – mobile health, Hep B – hepatitis B, ICCM – integrated community case management, SMS – short message service, TB – tuberculosis

### Decision support for illness management

The most common mHealth approach focused on ensuring CHWs’ compliance to standards and guidelines for health services [22,34,35,40,42-44,46,47,52]. Most commonly, these applications involved use of an electronic algorithm for childhood illness management which aided in standardisation of treatment, rational use of medication and timely referral through a series of guided steps within the mobile application [42-44,47,51,52].

In a cluster RCT of paper vs electronic algorithm for assessment of childhood illness conducted in Tanzania. Results showed that the use of electronic tool by CHWs vs paper led to a significant increase in children checked for danger signs (41% vs 74%, *P* = 0.04).and fewer prescription of antibiotics (70%, range 60%-85% in the control; 26%, range 14%-37%, adjusted risk ratio (aRR) 0.4 (95% CI = 0.2-0.6) in the paper; and 25%, range: 17%-33%, aRR 0.3 (95% CI = 0.2-0.5) in the electronic arm) [52].

Similarly, another cluster RCT conducted in Southwest Niger explored the use of a smartphone application by CHWs to support quality case management of children under five years of age presenting with diarrhoea, malaria, and pneumonia and to provide timely clinical data. The mHealth equipped CHWs showed a 3.4% higher QoC score (mean difference of 0.83 points) *P* = 0.009 with appropriate referrals (Mean QoC score of 2.7 in intervention vs 2.8 in the control group) and treatment scores (Mean QoC score of 8.3 in intervention vs 8.4 in the control group) similar to controls [34].

A quasi-experimental study conducted in Senegal to determine adverse event (AE) reporting of chemoprevention through smartphone application usage by CHWs divided health posts, into three arms; national system, enhanced spontaneous reporting (completed by physicians or nurses at health facilities using mobile phones) and active surveillance (completed by nurses at health posts and by CHWs with active follow-up of children at home). Results showed that the incidence of reported AEs was 2.4 using the national system, 30.6 using enhanced spontaneous reporting, and 21.6 using active surveillance per 1000 children treated per month [40].

Another quasi-experimental study in Malawi comparing adherence to iCCM guidelines by CHWs using mHealth- and paper-based tools demonstrated increased quality of care (QoC) in under five children with pneumonia, diarrhoea, and malaria whereby 80% of the CHWs in intervention group using the mHealth tool managed illnesses according to a gold standard as compared to 50% in control group [41].

In a study of development of an mHealth-based severity assessment, triage, treatment, and follow-up recommendation platform for use by CHWs with 2-60 month-old children, initial validation, usability, and acceptability testing was performed by comparing clinical assessments by CHWs with those of standard (health care professionals-HCPs). Results showed an 84% and 99% correlation between CHW generated assessments and those conducted by HCPs [42].

Not all studies demonstrated statistically significant differences between control and intervention groups, eg, a pilot RCT in Uganda, determined the impact of training of CHWs about under five pneumonia using educational videos on mobile tablets. Results showed intervention improved by 3.2/24 points and control 2.6/24 points, *t* = 1.15, *P* = 0.254 [35].

In a pre-trial implementation study in Uganda, mobile phone enabled software was developed aiming to improve competence of CHWs on Integrated Community Case Management(iCCM) and to strengthen reporting of data on danger signs of acutely ill under five-children [43]. It showed that the CHWs were able to master the required technology to improve provision of services to children in their village and expedite referral to appropriate levels of care [43].

The impact of this study was assessed in a subsequent observational study in Uganda through quantitative measures and qualitative evaluation using household surveys, in-depth interviews, and focus group discussions [44]. Results showed that 92.6% of acute cases were correctly managed and gains were shown in treatment planning apart from supply management and logistical efficiency.

A qualitative study in Ghana showed that an mHealth tool with an integrated digital version of IMCI algorithm and a software-based breath counter and pulse oximeter for pneumonia management appeared to help CHWs in correct diagnosis and treatment of children. Challenges included electricity requirements for charging and the increased time needed to complete the application [46].

Another qualitative study conducted in Burkina Faso in the context of a large-scale implementation of an electronic (e-IMCI) tool used by CHWs revealed a high level of satisfaction with the tool and that CHWs perceived the tool to be improving patient management and rational use of antibiotics [47].

Similarly, in another qualitative study evaluating impact and acceptability of an iCCM based mHealth tool used by CHWs, results showed that there was preference for usage of the mHealth tool as compared to paper-based tools [51].

A cohort study in Nepal evaluated a community-based surveillance system in which CHWs used mHealth technology to record diarrhoeal diseases and acute respiratory infections. The authors categorized the transformed community health rates by tertiles: low, medium, and high. Results showed that for diarrhoea, there were significant differences between low vs high (*P* = 0.001) and medium vs high (*P* = 0.04) tertiles in post-hoc comparisons between hospital and CHW rates whereas for ARI, the only significant difference was between low vs high (*P* = 0.01) [36]. They concluded that there was a modest correlation between hospital and community data and that use of mobile phones by CHWs might be a useful adjunct to other health care related and community related data sources for surveillance.

### Text message reminders

The second most common approach was the use of text messages as an adjunct to regular management of an illness or management using a mHealth application. The text messages focused mainly on reminders sent to the CHWs on household visits, and to caregivers for their children’s follow-up visits and adherence to treatment.

A cluster RCT in Kenya demonstrated that correct artemether-lumefantrine (AL) management improved by 23.7% (95% confidence interval (CI) = 9.0-33.7, *P* = 0.0007) immediately after the intervention and by 24.5% (95% CI = 11.6-35.7, *P* = 0.0001) six months after the intervention when text message reminders were sent to the CHWs on following national malaria management guidelines [53]. Authors of the same study also determined the cost-effectiveness of the use of these text message reminders on adherence to national malarial treatment guidelines and demonstrated the cost per additional child correctly managed was US$0.50 under study conditions, US$0.36 if implemented by the ministry of health in the same study locations under routine, and USD 0.03 if implemented nationally after being scaled-up [37].

Another RCT in Uganda on use of mHealth for diagnosis of TB by CHWs on general public showed that for children under five, yield of clinically and biologically confirmed cases was 5.2% in the intervention arm as compared to 3.8% in the control arm upon sending SMSs on sputum test results and/or follow up instructions [38].

An open label RCT was conducted in Kenya to test additional effects of SMS reminders on caregivers’ adherence to AL therapy with return to the health facility Results showed that, all AL doses were completed for 97.6% (282/289) of children in the control and 97.8% (267/273) in the intervention group (odds ratio (OR) = 1.10; 95% CI = 0.37-3.33; *P* = 0.860). Sending SMS reminders significantly increased odds of children returning to the facility on day 3 (81.4 vs 74.0%; OR = 1.55; 95% CI = 1.15-2.08; *P* = 0.004) and on day 28 (63.4 vs 52.5%; OR = 1.58; 95% CI = 1.30-1.92; *P* < 0.001) [45].

In a quasi-experimental study in Lao People Democratic Republic where text messages were used as reminders to caregivers for timely immunization of children, the difference in Hepatitis B vaccination coverage improved over the time of the intervention by57% (interquartile range (IQR) = 32%-88%, *P* < 0.0001) in the intervention districts as compared to control districts (20%, IQR = 0%-50%, *P* < 0.0001) [48].

A qualitative study in Kenya after this RCT on the impact of text message reminders to caregivers of children with malaria showed that there is direct benefit of use of text message reminders to caregivers to administer medication for under five children with malaria timely coupled with messages on completion of course and following standard treatment guidelines [49].

In another qualitative study conducted in Malawi on text message reminders for remembering treatment dates of children with malaria, reminders were reported to be a ‘quick’ and ‘easy’ way of communicating with the caregivers directly [39].

### Electronic health records

A third approach of use of mHealth was electronic health records (EHR) focusing on collection of patient data through mobile phone applications with subsequent generation of reports.

An RCT study conducted in China on use of a mobile application by village doctors to record vaccination status, make appointments, track children, and provide education to caregivers demonstrated that there was a positive behaviour change among caregivers. This was shown by a significant increase in full vaccination coverage from baseline to end-line in intervention (67% (95% CI = 58%-75%) to 84% (95% CI = 76%-90%), *P* = 0.028) and control groups (71% (95% CI = 62%-79%) to 82% (95% CI = 74%-88%), *P* = 0.014) [39].

A study conducted in Kenya on micro planning for a measles rubella campaign showed that data collection through a mobile application was time efficient as CHWs were able to collect data on three million children from 46 counties within four weeks using standard data collection forms incorporated within a mobile application [50].

### Demonstration of behaviour change and use of behaviour change models for intervention design

There were only three studies which reported behaviour change, for instance, the pre-implementation study in Uganda on use of mobile application as decision support, where behaviour change was demonstrated in the form of improved care seeking by caregivers due to the establishment of better relationships between caregivers and the CHWs [44]. Similarly, RCTs in Kenya and Uganda on text message reminder on AL and TB management respectively, demonstrated behaviour change by caregivers in terms of timely follow up and administration of antimalarial medication to children [38,53].

It was also observed that during the design of these interventions none of the studies demonstrated used of any behaviour change models or the use of any theoretically informed frameworks for classifying behaviour change within these interventions.

## DISCUSSION

This systematic review was conducted to explore current CHW based mHealth approaches for management of common infections in children under five with respect to inducing behaviour change and found 23 articles. The results showed that most of the approaches have used mobile applications to improve diagnosis through decision support, text messaging for education and/or reminders, and electronic health recording. Thus, the focus of these studies was more on health care service delivery and education about recognising or managing illness rather than prevention. The majority of the studies were also not clearly able to demonstrate behaviour change as most did not measure the behaviours targeted in the intervention. This has serious consequences as it prevents assessment of the effects of the intervention on the targeted behaviours that are expected to lead to health benefits. Additionally, the quality of the evidence available in these articles was not of high quality with only two studies covering all aspects of quality as assessed by the reviewers.

**Figure 2:**
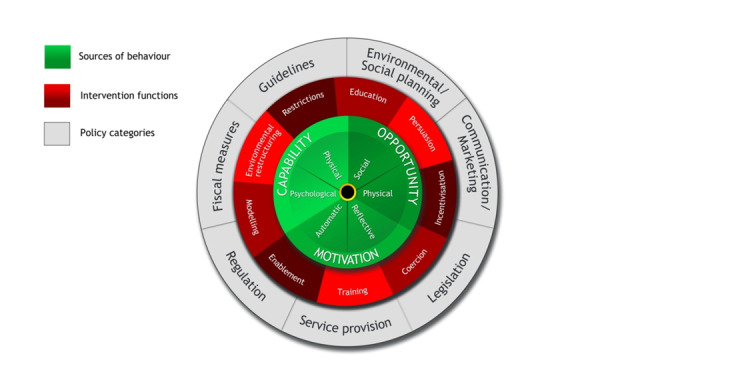
Behaviour change wheel [54].

**Figure 3:**
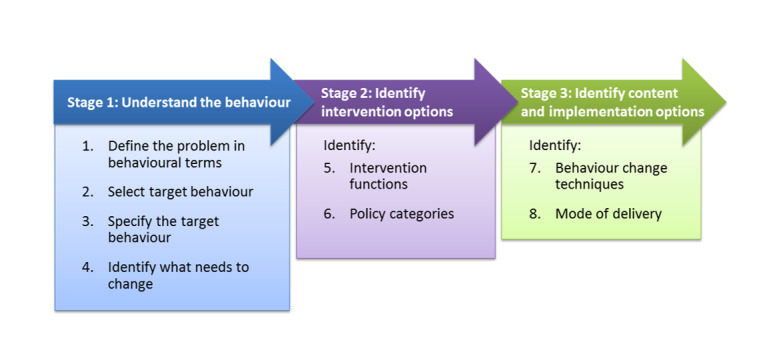
Stages of designing behaviour change interventions [54].

Developing and implementing interventions to change behaviour can be challenging [26]. Evidence has shown that intervention development usually starts without a systematic method and without drawing on any theories of behaviour change. Instead, personal experiences or a superficial analysis of the subject may be used as the starting point for intervention design, compromising the desired effects of the intervention [54]. One way of systematically characterising interventions enabling their outcomes to be linked with actions is the Behaviour Change Wheel (BCW) [26]. It has nine intervention functions and seven policy categories linked to the Capability-Opportunity-Motivation Behaviour (COM-B) model a model of behaviour at the hub of the wheel as shown in **Figure 2**. This allows interventions to be designed with the target behaviour in focus. Using this wheel, one can design an intervention through three stages; understanding of the behaviour to be targeted, identify intervention options to induce behaviour change and identify content and implementation options as shown in **Figure 3** [54]. Considering mobile health is gaining momentum due to extensive mobile network coverage, it can be used as a useful tool for inducing behaviour change [55]. There is suggestive evidence of the benefit of using mobile applications by CHWs coupled with tailored text message reminders being an effective delivery channel for positive behaviour change through its wide population reach and instant delivery [56-58]. However, for design of effective behaviour change interventions, there is a need for an underpinning framework that incorporates understanding of the nature of the behaviour to be changed, and an appropriate mechanism for characterising components of an intervention that can make use of this understanding [26].

For successful outcomes, establishment of long lasting and fruitful partnerships between users and policymakers throughout the process of the project is extremely crucial. Some of the good examples whereby policymakers or ownership at national level was established include studies from Ghana and Kenya [46,53]. In addition to this, culturally specific interventions are important as poorly designed non-specific campaigns seem to have a negative impact, especially in terms of language and literacy barriers [59,60].

The major strengths of our review include the robust data extraction across several databases, inclusion of articles from 1990, a period when mHealth was initiated globally [29]. An additional strength is the focus on under five children as compared to other such reviews. Limitations of the study include only drawing on English language papers and those that were peer reviewed. Although it is known that CHWs have been engaged in various mHealth based projects, however, our analysis of only 23 articles is not likely to represent this full range of projects. Additionally, the projects reported might not reflect negative results as those are not usually published [61]. As such, this review may be biased toward more positive results. Further, due to the heterogeneity of methods and design approaches in this evolving field of research, a meta-analysis was not feasible.

## CONCLUSION

Coupling of mobile technology with CHWs has the potential to benefit communities in improving management of illnesses in children under-five. High quality evidence of impact of such interventions on behaviour is relatively sparse and further studies should be conducted using theoretically informed frameworks/models of behaviour change.

## Additional material

Online Supplementary Document
