# Supplementary material for: Community health worker-based mobile health (mHealth) approaches for improving management and caregiver knowledge of common childhood infections: A systematic review
Source: J Glob Health. 2020 Dec 19;10(2):020438. doi: 10.7189/jogh.10.020438 (PMC7774026; doi:10.7189/jogh.10.020438)
Supplement: Online Supplementary Document [file jogh-10-020438-s001.pdf]

Appendix S1. SEARCH TERMS

#1 Age Specific terms

Child* OR Neonate* OR newborn* OR baby* OR Infant* OR Caregiver* OR carer* OR volunteer* OR mother* OR Father* OR grandparent* OR Childcare OR Parent*

#2 Disease Specific terms

Acute respiratory infection* OR Respiratory tract infection* OR Pneumonia* OR Respiratory diseases OR bacterial pneumonia OR Viral pneumonia OR Bronchopneumonia OR Infection* OR upper respiratory tract infection OR Lower respiratory tract infection* OR Viral Upper Respiratory tract infection* OR Diarrhea* or Diarrhoea OR Dengue OR Dengue fever OR Malaria* OR Measles OR Meningitis OR neonatal meningitis OR Typhoid OR Typhoid fever OR Tuberculosis OR TB OR Hepatitis OR Hepatitis A

#3 Mobile Health Based:

E-health OR ehealth OR Mobile OR mobile-phone OR M-health OR mhealth OR mobile-health OR Mobile healthcare OR Cell-phone* OR cell phone* OR Smart phone* OR smartphone* OR Smart-phone* OR Handheld OR tablet*OR mobile tablet* OR Telecommunication OR telecommunication in healthcare OR wireless OR App OR apps OR mobile-app* OR mobile telephone* OR mobile app* OR mobile app* in healthcare OR mhealth app* OR mobile healthcare app*OR Telemedicine OR tele-medicine OR Telehealth OR tele-health OR telecare OR Online OR on line OR internet OR Short Message service OR SMS OR Text-messag* OR text message OR text messaging OR Message reminder * OR Voice-messag* OR Voice message

#4 Community Health Worker Based:

Community-Health-Worker* OR CHW OR Lady Health Worker* OR LHW OR health extension worker* OR health-extension-worker* OR Frontline-health-worker OR Frontline health worker* OR Frontline health adj2 worker* OR Frontline Healthcare Provider* OR community health nurse* OR community-health-nurse* OR peer counsellor* OR peer health worker* OR community health volunteer* OR volunteer* OR promotora

Appendix S2. Electronic Search Strategy- Global Health Database

Database: Global Health <1973 to 2019 Week 44>
Search Strategy:

1child*.mp. (359549)
2child care/ or exp children/ (281801)
3neonate*.mp. (39012)
4exp neonates/ or exp infants/ (107841)
5newborn*.mp. (39620)
6baby*.mp. (12033)
7infants/ or exp neonates/ or exp children/ (357414)
8infant*.mp. (138581)
9caregiver*.mp. (8225)
10careproviders/ or exp child careproviders/ (2118)
11exp children/ or exp parents/ (302150)
12carer*.mp. (1550)
13volunteer*.mp. (28483)
14exp volunteers/ (8794)
15mother*.mp. (76702)
16exp mothers/ or exp parents/ or exp fathers/ (38809)
17father*.mp. (7053)
18grandparent*.mp. (774)
19exp grandparents/ (305)
20parent*.mp. (75001)
21exp parent care/ (54)
22acute respiratory infection*.mp. (2492)
23exp respiratory diseases/ or exp lower respiratory tract infections/ or exp pneumonia/ or exp upper respiratory tract infections/ (120570)
24respiratory tract infection*.mp. (11905)
25exp lower respiratory tract infections/ or exp bronchopneumonia/ (6877)
26exp upper respiratory tract infections/ (1968)
27pneumonia*.mp. (64790)
28exp bacterial pneumonia/ or exp pneumonia/ (25260)
29pneumonia/ (17024)
30bacterial pneumonia*.mp. (6529)
31exp bacterial pneumonia/ (5994)
32viral pneumonia*.mp. (368)
33bronchopneumonia*.mp. (1036)
34exp bronchopneumonia/ or exp lower respiratory tract infections/ (6877)

35broncho-pneumonia*.mp. (13)
36infection*.mp. (1009770)
37exp infection/ (73081)
38viral upper respiratory tract infection*.mp. (110)
39exp upper respiratory tract infections/ (1968)
40diarrhea*.mp. (37006)
41diarrhoea*.mp. (41391)
42exp diarrhoea/ (30218)
43dengue*.mp. (19312)
44exp dengue/ (14213)
45dengue fever.mp. (4660)
46malaria*.mp. (76159)
47exp malaria/ (57595)
48measles*.mp. (11654)
49exp measles/ (7770)
50meningitis*.mp. (17616)
51exp bacterial meningitis/ or exp meningitis/ (12892)
52exp viral meningitis/ (845)
53exp neonatal meningitis/ (29)
54typhoid*.mp. (4848)
55exp typhoid/ or exp enteric fevers/ (3541)
56tuberculosis*.mp. (59350)
57exp tuberculosis/ (49465)
58TB.mp. (25386)
59hepatitis*.mp. (77746)
60exp hepatitis/ or exp hepatitis a/ (66721)
61hepatitis A.mp. (6296)
62e-health.mp. (309)
63ehealth.mp. (315)
64mobile*.mp. (21178)
65exp mobile telephones/ (2388)
66mobile-phone*.mp. (1926)
67mobile phone*.mp. (1926)
68mhealth.mp. (514)
69exp telemedicine/ or exp telecommunications/ (10069)
70m-health.mp. (91)
71mobile health*.mp. (503)
72mobile-health*.mp. (503)
73mobile healthcare*.mp. (14)
74cell-phone*.mp. (572)

75cell phone*.mp. (572)
76smart phone*.mp. (123)
77smartphone*.mp. (1042)
78smart-phone*.mp. (123)
79handheld*.mp. (597)
80tablet*.mp. (11078)
81mobile tablet*.mp. (11)
82mobile-tablet*.mp. (11)
83telecommunication*.mp. (1380)
84exp telecommunications/ (9389)
85telecommunication in health*.mp. (0)
86telecommunication in healthcare*.mp. (0)
87wireless.mp. (477)
88app.mp. (1640)
89apps.mp. (533)
90mobile-app*.mp. (454)
91mobile app*.mp. (454)
92mobile app in health*.mp. (0)
93mobile app in healthcare*.mp. (0)
94telemedicine*.mp. (1529)
95exp telemedicine/ (1204)
96tele-medicine*.mp. (11)
97telehealth*.mp. (381)
98tele-health*.mp. (11)
99telecare.mp. (35)
100online.mp. (14612)
101exp on line/ (667)
102exp internet/ (8470)
103short message service*.mp. (260)
104sms.mp. (962)
105text-message*.mp. (747)
106text message*.mp. (747)
107text messaging.mp. (782)
108message reminder*.mp. (103)
109voice-message*.mp. (20)
110voice message*.mp. (20)
111community health worker*.mp. (2737)
112exp health care workers/ or exp community health workers/ (61212)
113community-health-worker*.mp. (2737)
114exp community health workers/ or exp health care workers/ or exp health extension workers/ (61212)

115CHW*.mp. (1361)
116lady health worker*.mp. (88)
117LHW*.mp. (99)
118health extension worker*.mp. (251)
119health-extension-worker*.mp. (251)
120frontline health worker*.mp. (112)
121(frontline health adj2 worker*).mp. [mP=abstract, title, original title, broad terms, heading words, identifiers, cabicodes] (130)
122frontline healthcare provider*.mp. (8)
123frontline-healthcare-provider*.mp. (8)
124community health nurse*.mp. (55)
125community-health-nurse*.mp. (55)
126peer counsellor*.mp. (37)
127peer counselor*.mp. (82)
128peer health worker*.mp. (11)
129community health volunteer*.mp. (196)
130exp volunteers/ (8794)
131promotora.mp. (106)
1321 or 2 or 3 or 4 or 5 or 6 or 7 or 8 or 9 or 10 or 11 or 12 or 15 or 16 or 17 or 18 or 19 or 20 or 21 (497288)
13322 or 23 or 24 or 25 or 26 or 27 or 28 or 29 or 30 or 31 or 32 or 33 or 34 or 35 or 36 or 37 or 38 or 39 or 40 or 41 or 42 or 43 or 44 or 45 or 46 or 47 or 48 or 49 or 50 or 51 or 52 or 53 or 54 or 55 or 56 or 57 or 58 or 59 or 60 or 61 (1192590)
13462 or 63 or 64 or 65 or 66 or 67 or 68 or 69 or 70 or 71 or 72 or 73 or 74 or 75 or 76 or 77 or 78 or 79 or 80 or 81 or 82 or 83 or 84 or 85 or 86 or 87 or 88 or 89 or 90 or 91 or 92 or 93 or 94 or 95 or 96 or 97 or 98 or 99 or 100 or 101 or 102 or 103 or 104 or 105 or 106 or 107 or 108 or 109 or 110 (57021)
13513 or 111 or 112 or 113 or 114 or 115 or 116 or 117 or 118 or 119 or 120 or 121 or 122 or 123 or 124 or 125 or 126 or 127 or 128 or 129 or 130 or 131 (96710)
136132 and 133 and 134 and 135 (262)
137gastroenteritis.mp. (11870)
138typhoid fever.mp. (2048)
139mobile device application*.mp. (7)
140mobile healthcare apps.mp. (0)
14122 or 23 or 24 or 25 or 26 or 27 or 28 or 29 or 30 or 31 or 32 or 33 or 34 or 35 or 36 or 37 or 38 or 39 or 40 or 41 or 42 or 43 or 44 or 45 or 46 or 47 or 48 or 49 or 50 or 51 or 52 or 53 or 54 or 55 or 56 or 57 or 58 or 59 or 60 or 61 or 137 or 138 (1194313)
14262 or 63 or 64 or 65 or 66 or 67 or 68 or 69 or 70 or 71 or 72 or 73 or 74 or 75 or 76 or 77 or 78 or 79 or 80 or 81 or 82 or 83 or 84 or 85 or 86 or 87 or 88 or 89 or 90 or 91 or 92 or 93 or 94 or 95 or 96 or 97 or 98 or 99 or 100 or 101 or 102 or 103 or 104 or 105 or 106 or 107 or 108 or 109 or 110 or 139 or 140 (57021)

143132 and 135 and 141 and 142 (263)
